# Neuroprotective Potential of Bone Marrow-Derived Mesenchymal Stem Cells Following Chemotherapy

**DOI:** 10.3390/biomedicines9070750

**Published:** 2021-06-29

**Authors:** Iman O. Sherif, Nora H. Al-Shaalan, Dina Sabry

**Affiliations:** 1 Emergency Hospital, Faculty of Medicine, Mansoura University, Mansoura 35516, Egypt; 2 Chemistry Department, College of Science, Princess Nourah Bint Abdulrahman University, Riyadh 11671, Saudi Arabia; nhalshaalan@pnu.edu.sa; 3 Medical Biochemistry and Molecular Biology Department, Faculty of Medicine, Cairo University, Cairo 11562, Egypt; dinasabry@kasralainy.edu.eg; 4 Medical Biochemistry and Molecular Biology Department, Faculty of Medicine, Badr University in Cairo, Badr City 11829, Egypt

**Keywords:** cisplatin, neurotoxicity, BM-MSCs, IL-6, caspase-3, Ki-67

## Abstract

Cisplatin (CP) is extensively used in the medical oncology field for malignancy treatment, but its use is associated with neurological side effects that compromise the patients’ quality of life. Cytotherapy is a new treatment strategy for tissue damage that has recently emerged. The use of bone marrow-derived mesenchymal stem cells (BM-MSCs) was investigated for its therapeutic potential against CP-induced chemobrain as well as various models of brain damage. This study was carried out to elucidate, for the first time, the role of the intravenous injection (IV) of BM-MSCs against CP-induced neurotoxicity in a rat model through investigation of the parameters of oxidative stress, inflammation, and apoptosis in brain tissue. A rat model of neurotoxicity was generated by intraperitoneal injection of 7.5 mg/kg CP while 2 × 10^6^ BM-MSCs was given by IV as a therapeutic dose. Injection of CP led to a significant rise in malondialdehyde and nitric oxide levels accompanied by a marked depletion of superoxide dismutase and reduced glutathione content in brain tissue in comparison to the normal control (NC) rats. Furthermore, a remarkable rise in the brain levels of inflammatory cytokines interleukin (IL)-1β and IL-6, together with the expression of apoptotic marker caspase-3, and the downregulation of the brain expression of proliferating marker Ki-67 in brain tissue were detected in the CP group compared to the NC group. Histopathological alterations were observed in the brain tissue of the CP group. BM-MSCs mitigated the biochemical and histopathological alterations induced by CP without affecting brain cell proliferation. BM-MSCs could be used as a promising neuroprotective agent against CP-induced neurotoxicity.

## 1. Introduction

Chemotherapy is the current most effective strategy to combat cancer, which is considered one of the leading causes of death worldwide [1,2]. The first antineoplastic agent, belonging to the family of platinum-based chemotherapy, that was approved in 1978 by the FDA is cisplatin (CP). It exhibited great beneficial effects towards a variety of cancers, including solid tumors [3]. Despite its effectiveness, CP displayed adverse effects leading to toxicity to various organs, which limited its clinical application [3,4]. Cisplatin-induced neurotoxicity emerged as a drawback of using CP in experimental and clinical studies [5,6].

Identifying the possible mechanisms underlying CP-induced neurotoxicity will aid researchers in finding a suitable management strategy for this deleterious effect; however, so far, the exact molecular mechanism of the neurotoxicity induced by CP has still not been fully explored [5]. It was reported that the CP-induced organ toxicity was due to reactive oxygen species (ROS) generation and the disturbance of the antioxidant defense system. Moreover, CP is well known to have a DNA-damaging effect due to the induction of apoptosis [3].

Effective therapeutic agents are needed to overcome the neurotoxicity induced by chemotherapy [1]. Different strategies have been used to ameliorate the adverse effects of CP, including its combination with other drugs, either chemical or natural products [6,7]. However, recent advances in regenerative medicine have focused on mesenchymal stem cells (MSCs) as a therapeutic option for various types of organ toxicity induced by chemotherapy [8,9,10,11] as well as different models of brain injury [12,13,14].

MSCs are easily obtained from bone marrow and able to stimulate neuronal growth, suppress apoptosis, decrease free radical levels, and regulate inflammation through their paracrine actions [2]. Human MSCs showed a neuroprotective effect against CP neurotoxicity via protecting dorsal root ganglia [15]. Moreover, nasal administration of MSCs restored the brain damage and the cognitive impairment induced by CP [1]. Therefore, our study was carried out to investigate, for the first time, the role of intravenous injection (IV) of bone marrow-derived MSCs (BM-MSCs) against CP-induced neurotoxicity by investigating the oxidative, inflammatory, apoptosis, and proliferation biomarkers.

## 2. Materials and Methods

### 2.1. Chemicals and Reagents

Malondialdehyde (MDA), nitric oxide (NO), superoxide dismutase (SOD), and reduced glutathione (GSH) determination kits were purchased from Biodiagnostic (Giza, Egypt) with Cat. No.; MD 25 29, NO 25 33, SD 25 21, and GR 25 11, respectively. Meanwhile, interleukin-6 (IL-6, Cat. No. CSB-E04640r) and IL-1β (Cat. No. CSB-E08055r) ELISA kits were purchased from Cusabio Biotech Co., Wuhan, China.

For immunohistochemistry, the following primary antibodies were purchased: caspase-3 (1:100; Invitrogen, Carlsbad, CA, USA, Cat No. PA5-77887) and Ki-67 (1:100; Dako, Carpinteria, CA, USA, Cat No. M7248). However, the secondary antibodies used were a goat anti-rabbit secondary antibody (Envision + System HRP-labeled polymer, Cat No. K4003) for caspase-3 detection in addition to the mouse secondary antibody (Envision + System HRP-labeled polymer, Cat No. K3468) for Ki-67 detection, and they were provided by Dako, Carpinteria, CA, USA.

Phosphate-buffered saline (PBS), fetal bovine serum (FBS), and Dulbecco’s modified Eagle’s medium (DMEM) were provided by Gibco BRL, Waltham, MA, USA. The StemPro^®^adipogenesis (Cat No. A1007001), osteogenesis (Cat No. A1007201), and chondrogenesis (Cat No. A1007101) differentiation kits were purchased from Gibco, Life Technology, Carlsbad, CA, USA. Meanwhile, Oil Red O, Alizarin Red S, and Alcian blue stains were provided by Sigma-Aldrich, St Louis, MO, USA.

### 2.2. Preparation of BM-Derived MSCs

The BM cells were harvested by flushing the rats’ tibiae and femurs with PBS supplemented with 10% FBS. Then, the nucleated cells were isolated, resuspended in complete culture DMEM supplemented with 1% penicillin–streptomycin, and incubated in 5% humidified CO_2_ at 37 °C for 12–14 days as primary culture until the formation of large colonies (80–90% confluence). Then, the cultures were washed two times with PBS at 37 °C, and the cells were trypsinized with 0.25% trypsin in 1mM EDTA for 15 min. After centrifugation at 300× *g* for 5 min, cells were resuspended with serum-supplemented medium and incubated in a 50 cm^2^ Falcon culture flask. The resulting cultures were denoted as first-passage cultures and expanded until passage three in vitro [16].

### 2.3. Characterization of BM-Derived MSCs

The MSCs in culture were characterized by their morphology, adherence, differentiation, and surface markers. Fluorescent Activated Cell Sorting (FACS) was used to assess the positivity of CD90 and the negativity of CD34. Furthermore, the osteocyte differentiation was accomplished by the osteocyte differentiation kit and stained with Alizarin Red S stain, while the adipocyte differentiation was detected using the adipocyte differentiation kit and they were stained with Oil Red O stain. Finally, the chondrocyte differentiation was achieved using the chondrogenic differentiation kit and stained with Alcian blue stain.

### 2.4. Experimental Protocol

Male Sprague–Dawley rats, weighing 200–250 g, were used in our experiment. Rats were permitted free access to food and tap water while kept under a 12 h dark/light cycle at 25° ± 2 °C. The animal protocol was approved by local Ethical Committee at Mansoura University, Egypt and the handling of the experimental animals used was in accordance with the Care and Use of Laboratory Animals guide (NIH publication No. 85-23, revised 2011).

Rats were randomly divided into three groups with 8 rats/group:

1. Normal control group (NC): animals were injected with PBS intraperitoneally (ip).
2. Cisplatin group (CP): animals were injected with CP ip at a dose of 7.5 mg/kg (Hospira, Warwickshire, UK) to induce neurotoxicity [4].
3. Stem cell group (BM-MSCs): animals were injected with CP (7.5 mg/kg, ip) and, on the next day, they were injected intravenously with 2 × 10^6^ BM-MSCs [9].

### 2.5. Collection of Samples

Sample collections were carried out after 7 days, during which rats were anesthetized and sacrificed. Brain tissues were dissected immediately and weighed. Then, one half of the brain was homogenized in ice-cold PBS and the homogenates were centrifuged at 4 °C, 3000 rpm for 15 min, and were kept at −80 °C until used for subsequent biochemical assessment. The other half of the brain tissue was immersed in formalin solution for histopathological and immunohistochemical examinations.

### 2.6. Histopathological Evaluation

Brain tissues were immersed in formalin 10% for 48 h, dehydrated, and then embedded in paraffin blocks. Briefly, 5 μm sections were cut and left in an oven for 20–30 min and deparaffinized by placing them in xylene for 20–30 min, rehydrated in alcohol (absolute, 90%, 70%) for 1–2 min each, and washed with running tap water for 5 min; then, they were stained with hematoxylin (H), and the slides were incubated for 5 min at room temperature. Next, they were washed with running tap water and immersed in 1% HCl for 1 s, and they were then washed with running tap water until a blue color appeared. Further, slides were incubated in eosin (E) for 30 s to 2 min and then washed with running tap water; after this, they were immersed in alcohol (70% for 1 min, 90% for 1 min, absolute for 5 min) and then immersed in xylene. Slides were covered and then examined under a light microscope by a blinded pathologist.

### 2.7. Estimation of Oxidative and Nitrosative Stress Markers

To determine the oxidative and nitrosative stress in animals’ brain tissue homogenate, oxidative stress parameter MDA and nitrosative stress indicator NO were measured colorimetrically by using available kits, following the manufacturer’s instructions. The MDA was estimated by using thiobarbituric acid (TBA) and its values were expressed as nmol/g tissue [17]; however, NO activity was measured as previously described [18], in which nitrites (NO^−2^) were measured as an index of NO production by using Griess reagent and its values were expressed as μmol/g tissue. The results were presented as fold change relative to NC group.

### 2.8. Determination of the Antioxidant Activity

The antioxidant activity in the brain tissue of animals was evaluated spectrophotometrically by measuring the antioxidant levels of SOD and GSH in the brain tissue homogenate using available kits, following the manufacturer’s instructions. GSH activity was measured as described before [19] and its values were expressed as mmol/g tissue, while the activity of SOD was estimated according to a previous method [20] and its values were expressed as U/g tissue. The results were presented as fold change relative to NC group.

### 2.9. Determination of the Inflammatory Parameters

The brain levels of the proinflammatory cytokines were determined by using IL-6 and IL-1β ELISA kits in the brain tissue homogenate, following the manufacturer’s instructions. The values were expressed as pg/mg protein and the results were presented as fold change relative to % NC group.

### 2.10. Immunohistochemistry (IHC) Examination for Apoptotic and Cell Proliferation Biomarkers

Other sets of brain paraffin blocks were deparaffinized and rehydrated. For antigen retrieval, slides were heated for 10 min in a solution of 10 mM citrate buffer (pH 6). Incubation with primary antibodies against either the apoptotic marker caspase-3 or cell proliferation marker Ki-67 was carried out and followed by incubation with the appropriate secondary antibodies. Finally, slides were visualized by adding diaminobenzidine tetrahydrochloride (DAB) and counterstaining with Mayer’s hematoxylin; then, they were examined under a light microscope by a blinded pathologist.

The labeling index for ki-67 was calculated as the % of positively stained cell/total 1000 cells in one field. A cell was considered positive when it had a stained brawn nucleus. However, for IHC, the intensity scores of caspase-3 were assessed using a scoring scale with values of 0, 1, 2, and 3, which refer to negative, weak, moderate, and strong staining, respectively.

### 2.11. Statistical Analysis

Results were presented as mean ± SD. Significant differences between groups were evaluated using one-way ANOVA followed by a post-hoc Bonferroni test with the use of SPSS version 20 (Chicago, IL, USA). Moreover, GraphPad Prism version 6 (San Diego, CA, USA) was used for the statistical analysis of the histopathological scores. Kruskal–Wallis test followed by Dunn’s test was used for IHC score statistical analysis of caspase-3; meanwhile, one-way ANOVA followed by Tukey’s test was used for Ki-67 immunolabeling statistical analysis. When *p* was <0.05, statistical significance was considered.

## 3. Results

### 3.1. Characterization of BM-MSCs

**Figure 1:**
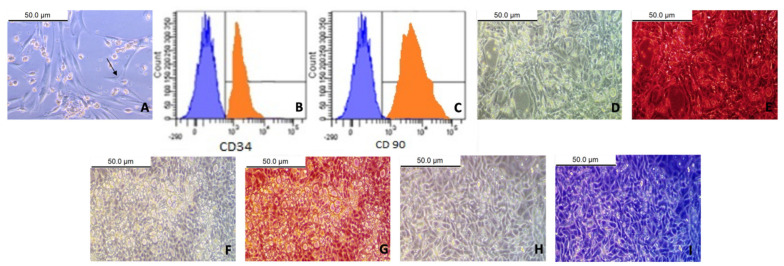
Bone marrow-derived mesenchymal stem cells (BM-MSCs) were distinguished by: fusiform shape (black arrow) (**A**); FACS: cells were uniformly negative for CD 34^−^ and weakly expressed (1.8%) (**B**) and uniformly positive for CD90^+^ and strongly expressed (98.2%) (**C**); Differentiation: MSCs differentiated into osteocytes and presented before staining (**D**) and after staining with Alizarin red (**E**) (×40); MSCs differentiated into adipocytes and presented before staining (**F**) and after staining with Oil Red O (**G**) (×40); MSCs differentiated into chondrocytes and presented before staining (**H**) and after staining with Alcian blue (**I**) (×40).

As presented in Figure 1, the cultured BM-MSCs were characterized by their adhesiveness and fusiform shape (A); FACS assessment of the negativity of CD34^−^ (B) and the positivity of CD90^+^ (C) specific to MSCs; and the differentiation of BM-MSCs into osteocytes (D and E), adipocytes (F and G), and chondrocytes (H and I).

### 3.2. BM-MSCs Injection Improved the Brain Histopathological Alterations in CP-Intoxicated Rats

**Figure 2:**
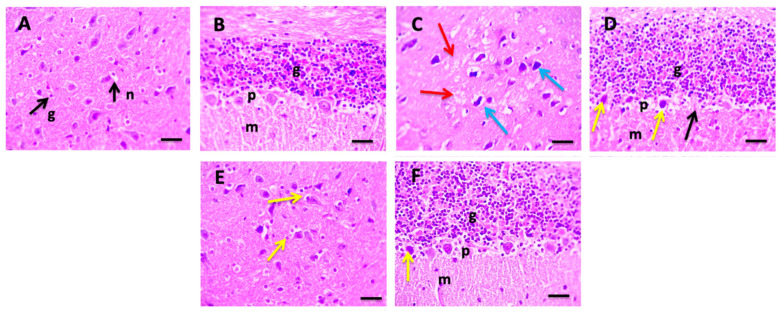
Microscopic pictures of H&E-stained cerebral sections in the normal control (NC) group (**A**) showing normal pyramidal neurons (n) and glial cells (g). Cerebellar sections showing normal grey and white matter in the NC group (**B**). Grey matter showing normal granular (g), Purkinje (p), and molecular (m) layers. Cerebral sections from cisplatin (CP) group (**C**) showing vacuolations (red arrows), marked shrinkage in pyramidal neurons, perineural edema with extensively dark pyknotic nuclei (blue arrows). Cerebellar sections from CP group (**D**) showing deeply stained Purkinje cells with pyknotic nuclei (yellow arrows) and loss of Purkinje nerve fibers (black arrows) (p). Cerebral sections from bone marrow-derived mesenchymal stem cells (BM-MSCs) group (**E**) showing satellitosis (yellow arrows). Cerebellar sections from BM-MSCs group showing decreased damage to Purkinje nerve fibers (**F**). ×400, bar 50 μm.

The neurotoxicity of CP as well as the neuroprotective potential of BM-MSCs was examined histopathologically, as illustrated in Figure 2. Normal cerebral and cerebellar sections were observed in the NC group (A and B); however, CP injection elicited pathological alterations in brain tissue, including vacuolations, a marked shrinkage in pyramidal neurons, perineural edema with extensively dark pyknotic nuclei, and loss of Purkinje nerve fibers (C and D). In contrast, cerebral and cerebellar sections treated with BM-MSCs exhibited a better brain architecture, with diminished damage to Purkinje nerve fibers (E and F).

### 3.3. BM-MSCs Injection Suppressed the CP-Induced Brain Oxidative and Nitrosative Stress in Rats

**Figure 3:**
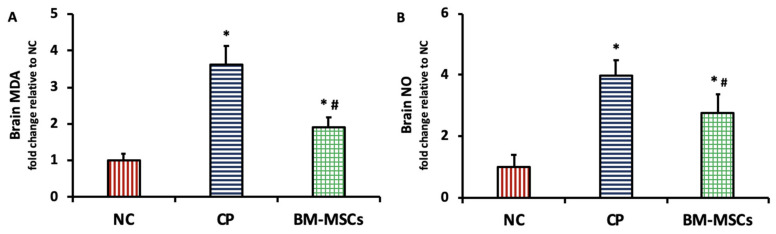
Impact of BM-MSCs injection on MDA (**A**) and NO (**B**) content in the brain tissue of CP-intoxicated rats. Data are presented as mean ± SD, *n* = 5. * *p* < 0.05 vs. NC group, ^#^ *p* < 0.05 vs. CP group. NC: normal control, CP: cisplatin, BM-MSCs: bone marrow-derived mesenchymal stem cells, MDA: malondialdehyde, NO: nitric oxide.

As seen in Figure 3, the injection of CP triggered a significant rise in the brain content of lipid peroxidation marker MDA (3.61 ± 0.53-fold, *p* = 0.000) (A) and NO (3.98 ± 0.51-fold, *p* = 0.000) (B) in comparison to the NC group. However, a significant decline in the brain levels of MDA (1.91 ± 0.25-fold, *p* = 0.000) and NO (2.75 ± 0.62-fold, *p* = 0.008) was observed in the group treated with BM-MSCs injection in comparison to the CP-injected group.

### 3.4. BM-MSCs Injection Boosted the CP-Induced Antioxidant Depletion in Rats

**Figure 4:**
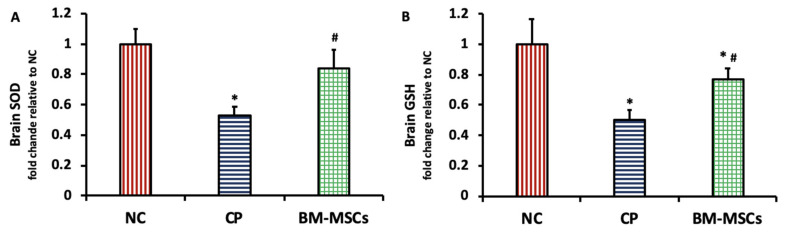
Impact of BM-MSCs injection on SOD (**A**) and GSH (**B**) in the brain tissue of CP-intoxicated rats. Data are presented as mean ± SD, *n* = 5. * *p* < 0.05 vs. NC group, ^#^ *p* < 0.05 vs. CP group. NC: normal control, CP: cisplatin, BM-MSCs: bone marrow-derived mesenchymal stem cells, SOD: superoxide dismutase, GSH: reduced glutathione.

As demonstrated in Figure 4, CP injection resulted in a marked depletion of the brain content of SOD (0.53 ± 0.06-fold, *p* = 0.000) (A) as well as brain GSH (0.49 ± 0.07-fold, *p* = 0.000) (B) levels in comparison to the NC group. On the other hand, rats treated with BM-MSCs showed restored brain antioxidant levels through a significant elevation of brain SOD (0.84 ± 0.12-fold, *p* = 0.002) and brain GSH (0.77 ± 0.07-fold, *p* = 0.008) levels when compared with CP-intoxicated rats.

### 3.5. BM-MSCs Injection Attenuated the CP-Induced Brain Inflammation in Rats

**Figure 5:**
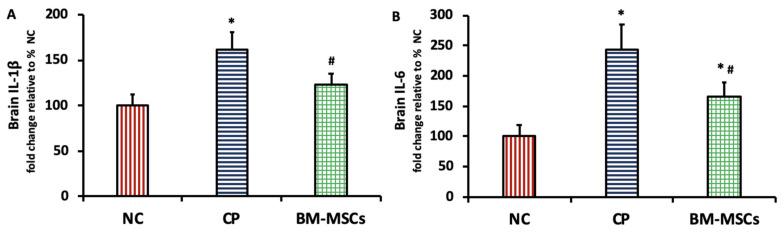
Impact of BM-MSCs administration on IL-1β (**A**) and IL-6 (**B**) in the brain tissue of CP-intoxicated rats. Data are presented as mean ± SD, *n* = 5. * *p* < 0.05 vs. NC group, ^#^ *p* < 0.05 vs. CP group. NC: normal control, CP: cisplatin, BM-MSCs: bone marrow-derived mesenchymal stem cells, IL: interleukin.

Figure 5 shows a marked elevation in the brain levels of the proinflammatory cytokines IL-1β (161.14 ± 19.77%, *p* = 0.000) (A) and IL-6 (243.6 ± 41.88%, *p* = 0.000) (B) after CP injection compared to the NC group. BM-MSCs led to a marked decline in the brain content of IL-1β (123.14 ± 11.75%, *p* = 0.005) and IL-6 (166.5 ± 22.94%, *p* = 0.004) when compared with the CP group.

### 3.6. BM-MSCs Injection Mitigated the CP-Induced Brain Apoptosis in Rats

**Figure 6:**
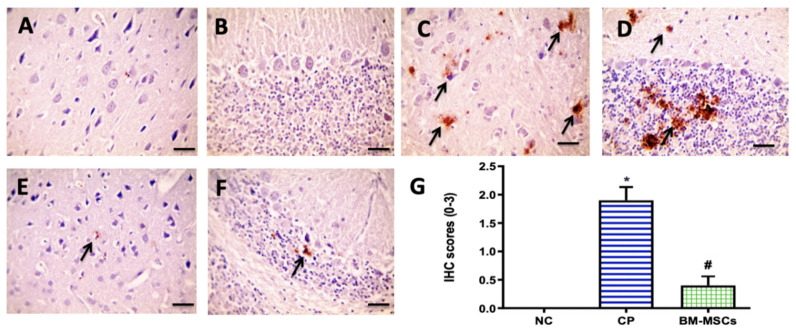
Microscopic pictures of immunostained cerebral (**A**) and cerebellar (**B**) sections against caspase-3 displaying negative staining in the NC group. Cerebral (**C**) and cerebellar (**D**) sections from CP group showing marked positive expression in neurons (black arrows). Meanwhile, cerebral (**E**) and cerebellar (**F**) sections from BM-MSCs group, showing very mild positive expression in neurons (black arrows). ×400, bar 50 μm. Statistical analysis of the IHC scores of caspase-3 expression in immunostained brain sections (**G**). * *p* < 0.05 vs. NC group, ^#^ *p* < 0.05 vs. CP group. NC: normal control, CP: cisplatin, BM-MSCs: bone marrow-derived mesenchymal stem cells.

The effect of BM-MSCs administration on the expression of apoptotic marker total caspase-3 protein was investigated, and the results are shown in Figure 6. Negative caspase-3 expression was seen in the NC group (A and B); however, marked positive brown expression was detected in the CP group (C and D). The BM-MSCs group exhibited very mild expression (E and F). Results of the statistical analysis of the IHC of caspase-3 expression in brain tissue are presented in Figure 6G, in which CP injection showed a marked rise in the IHC score (1.9 ± 0.23) in comparison to the NC group, while a significant decline in the IHC score was observed in the BM-MSCs group (0.4 ± 0.16) in comparison to CP, *p* < 0.05.

### 3.7. BM-MSCs Injection Did Not Affect the CP-Induced Downregulation of Brain Proliferation Marker Ki-67 in Rats

**Figure 7:**
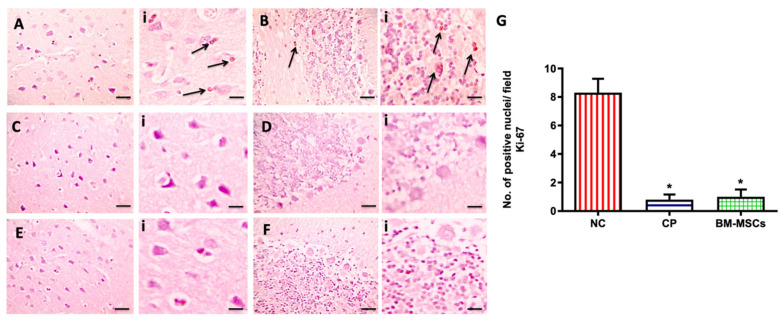
Microscopic pictures of immunostained sections from cerebral cortex (**A**) and cerebellum (**B**) against Ki-67, showing increased positive brown expression in cerebral cortex (**A**) and cerebellum (**B**) from NC group, markedly decreased positive brown expression in cerebral cortex (**C**,**E**) and cerebellum (**D**,**F**) from CP and BM-MSCs groups, respectively. Black arrows point to positive staining. The IHC counterstained with Mayer’s hematoxylin. ×400, bar 50 μm with its corresponding insert (i) ×1000. Statistical analysis of numbers of positive nuclei against Ki-67 in immunostained brain sections (**G**). * *p* < 0.05 vs. NC group. NC: normal control, CP: cisplatin, BM-MSCs: bone marrow-derived mesenchymal stem cells.

The proliferating protein marker Ki-67 was examined immunohistochemically in the brain tissue in all experimental groups, as shown in Figure 7. Marked positive brown Ki-67 protein expression was detected in the NC group (A and B), while remarkably decreased positive expression was observed in the CP (C and D) and BM-MSCs (E and F) groups. Statistical analysis of the numbers of positive nuclei against Ki-67 in immunostained brain sections (G) showed a significant decrease in the CP (0.8 ± 0.35) and BM-MSCs (1 ± 0.51) groups in comparison to the NC group (8.3 ± 0.97), *p* < 0.05.

## 4. Discussion

Cisplatin, similarly to other chemotherapeutic agents, is associated with neurotoxicity, which is considered one of the major adverse effects associated with chemotherapy [21]. Various underlying mechanisms of CP-induced neurotoxicity have been assumed. The first could be attributed to the disturbance of the oxidant/antioxidant balance as CP induces oxidative stress through increasing tissue oxidants and depleting tissue antioxidants [22]. Large quantities of NO have been found to be produced under oxidative stress by iNOS, with the subsequent formation of peroxynitrite, which causes a consequent damaging effect due to GSH level depletion and antioxidant cellular activity suppression [23]. After CP injection, our study presented a significant rise in the lipid peroxidation product MDA in addition to the NO content, along with a significant decline in GSH and SOD levels in the brain tissue, and these results were in line with those of other previous studies [6,24,25].

Interrelated mechanisms between oxidative stress and inflammation have been reported in the development of CP-induced neurotoxicity as CP induces ROS overproduction, which activates inflammatory signaling pathways and enhances the release of proinflammatory cytokines [21,25]. Therefore, the second possible factor that could be involved in the pathophysiological mechanism of CP-induced toxicity in brain tissue is neuroinflammation [21,25].

The NF-kB is a transcription factor that plays an essential role in inflammation in CP-induced neurotoxicity through the induction of proinflammatory cytokines [26]. Following CP injection, a significant elevation of the inflammatory cytokine concentrations of IL-1β and IL-6 in the brain tissue was detected in our study, confirming the presence of neuroinflammation. These findings are in line with those of other reports [21,24,25].

Furthermore, CP-induced oxidative stress in brain tissue triggers oxidative damage and apoptotic cell death [25,27]; therefore, apoptosis could be considered the third mechanism implicated in CP-induced neurotoxicity. The initial crucial step in apoptosis is caspase activation [26,27]. A marked upregulation of caspase-3 protein expression in brain tissue in the CP-injected rats in comparison to the control rats was recorded in our study. It was documented that the increased protein level and expression of the executive caspases such as caspase-3 in brain tissue following CP administration mediated the neuronal apoptotic programmed cell death [25,27].

It has been found that CP injection elicits many histopathological alterations in brain tissue [4,27,28]. Similarly, in our study, the cerebral and cerebellar tissue of the CP-injected group presented with vacuolations, a marked shrinkage in pyramidal neurons, perineural edema with extensively dark pyknotic nuclei, deeply stained Purkinje cells with pyknotic nuclei, and loss of Purkinje nerve fibers.

On the other hand, Ki-67 protein expression is strongly linked to cell proliferation and it is a common marker for the detection of newly synthesized cells in brain damage following injury [29]. We observed a marked reduction in Ki-67 protein expression in brain tissue in the CP group when compared to the controls. In line with this, an experimental study reported a marked reduction in Ki67 immunolabeled cells in rats’ brains after a single dose of 12 mg/kg CP ip was injected compared to control rats, concluding that CP could inhibit cell proliferation [30]. CP has been reported to trigger oxidative stress and inflammatory responses, both of which could be linked to neurogenesis suppression [30].

Cisplatin induced neurotoxicity as the neuronal cells are more vulnerable to oxidative stress because of their insufficient antioxidant defenses and raised lipid contents [31]. Therefore, possible interventions to mitigate the neurotoxicity induced by CP have been investigated through concurrent treatment with agents with antioxidant capability [24]. Moreover, inhibition of the inflammatory mediators and suppression of apoptosis could be targets for neuroprotection [24,25,27].

The neuroprotective potential of BM-MSCs has been reported previously in different animal models through either the intranasal route, as in CP-induced brain damage and cognitive impairment [1], or through the intravenous route in models including paclitaxel-induced neuropathy [32], STZ-induced diabetic neuropathy [33], and intracerebral hemorrhage [23], and these reports attributed the BM-MSCs neuroprotective action to their antioxidant, anti-inflammatory, and anti-apoptotic activities [23,32,33].

In the present study, BM-MSCs injection led to a marked attenuation of CP-induced oxidative brain damage through minimizing oxidative and nitrosative stress markers significantly, along with the elevation of antioxidant levels in the brain tissue. Our team has previously reported the antioxidant activity of BM-MSCs in a model of CP-induced testicular toxicity [9]. It has also been reported that MSCs could exhibit antioxidant activity by acting either directly through scavenging ROS or indirectly via raised antioxidant defenses [34].

Furthermore, the current work reported that BM-MSCs injection suppressed inflammation and apoptosis induced in brain tissue following CP administration via a significant reduction in the levels of brain proinflammatory cytokines IL-1β and IL-6 as well as the apoptotic marker caspase-3, confirming the anti-inflammatory and anti-apoptotic actions of BM-MSCs, which have been reported previously in models of CP-induced reno- and testicular toxicity [9,10]. A growing body of evidence shows that the anti-inflammatory and anti-apoptotic activities of MSCs are mediated through their inhibitory action on oxidative stress and ROS production [34].

It has been found that brain damage treatment either by nasal or cranial administration of MSCs does not integrate in the brain; however, MSCs have an endogenous repair mechanism that could improve brain function [1]. Moreover, it was reported in a model of CP-induced brain damage that nasal MSC administration following CP was not detected 3 days after CP administration, suggesting that MSCs could not transdifferentiate into neuronal cells [1]. Evangelista and his colleagues in 2018 reported during diabetic neuropathy that the beneficial effects of IV MSC injection are independent of the existence of a marked number of transplanted cells in the affected tissue [33].

Interestingly, our results showed that BM-MSCs injection could not upregulate the expression of proliferating marker Ki-67 protein in the brain tissue following CP administration this could be due to the fact that the dose, duration, or route of administration of the injected MSCs were not able to induce cell proliferation; therefore, further studies should be conducted to explore this.

It has been suggested that venous injection of MSCs could activate processes outside the brain, causing endogenous stem cells to be stimulated, which play a role in repairing the damage [35]. The results of the current study suggest that BM-MSCs could attenuate the CP-induced neurotoxicity via a paracrine mechanism through the secretion of many factors that contribute to the reduction of oxidative stress, inflammation, and apoptosis [34,35].

## 5. Conclusions

**Figure 8:**
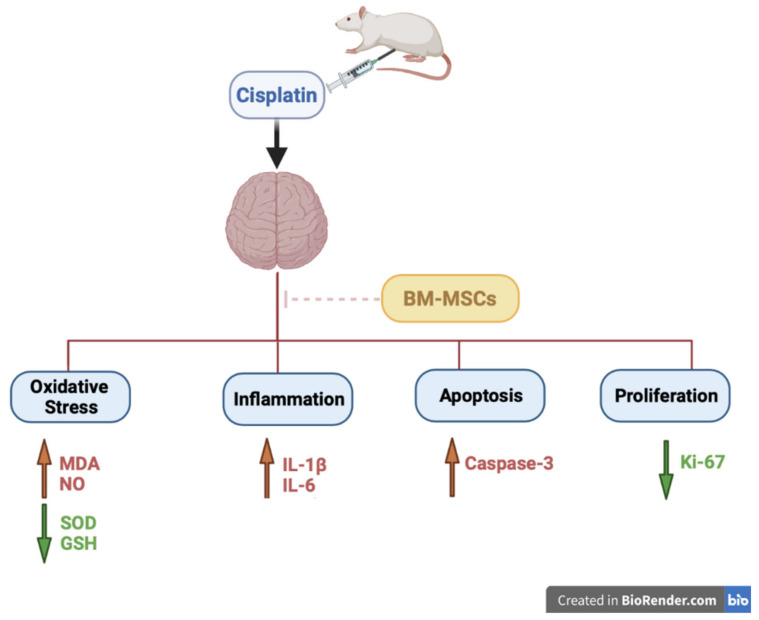
Schematic diagram illustrating the neuroprotective mechanism of BM-MSCs against cisplatin-induced neurotoxicity. BM-MSCs: bone marrow-derived mesenchymal stem cells, MDA: malondialdehyde, NO: nitric oxide, SOD: superoxide dismutase, GSH: reduced glutathione, IL: interleukin.

In conclusion, BM-MSCs could act as a neuroprotective agent in a model of CP-induced neurotoxicity through multiple mechanisms, as illustrated in Figure 8; (a) suppression of oxidative stress and restoration of antioxidant levels via decreasing brain MDA and NO content along with increasing brain SOD and GSH levels; (b) inhibition of brain inflammatory cytokines IL-1β and IL-6 levels; (c) minimizing apoptosis by downregulation of caspase-3 expression. Future clinical research should be conducted to validate the role of using BM-MSCs clinically alongside chemotherapy to mitigate the neurotoxicity in addition to elucidating in depth the neuroprotective molecular mechanism of BM-MSCs.

## Data Availability

All data generated during this study are included in the published manuscript.
